# Ferric chloride-induced murine carotid arterial injury: A model of redox pathology^☆^

**DOI:** 10.1016/j.redox.2012.11.001

**Published:** 2013-01-26

**Authors:** Wei Li, Thomas M. McIntyre, Roy L. Silverstein

**Affiliations:** a Department of Cellular and Molecular Medicine, Lerner Research Institute, Cleveland Clinic, 9500 Euclid Avenue, Cleveland, OH 44195, USA; b Department of Molecular Medicine, Cleveland Clinic Lerner College of Medicine of Case, Western Reserve University, Cleveland, OH, USA; c Department of Medicine, Medical College of Wisconsin, Milwaukee, WI, USA

**Keywords:** CA, carotid artery, Ferric chloride, Thrombosis, Carotid artery, Animal model

## Abstract

Ferric chloride (FeCl_3_) induced vascular injury is a widely used model of occlusive thrombosis that reports platelet activation in the context of an aseptic closed vascular system. This model is based on redox-induced endothelial cell injury, which is simple and sensitive to both anticoagulant and anti-platelets drugs. The time required for platelet aggregation to occlude blood flow gives a quantitative measure of vascular damage that is pathologically relevant to thrombotic disease. We have refined the traditional FeCl_3_-induced carotid artery model making the data highly reproducible with lower variation. This paper will describe our artifices and report the role of varying the oxidative damage by varying FeCl_3_ concentrations and exposure. To explore a maximum difference between experimental groups, adjustment of the selected FeCl_3_ dose and exposure duration may be necessary.

## Graphical abstract

**Graphical abstract:**

**Figure:**
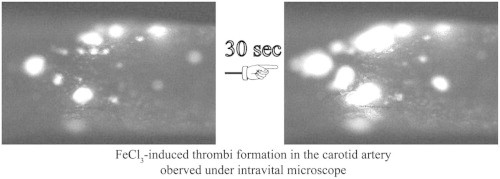

**Highlights:**

► Oxidative damage to large blood vessels is sufficient to induce thrombosis. ► Oxidative damage alone is a thrombotic stimulus. ► Different level of redox injury to the vessel wall induces varied grades of thrombosis. ► The time to occlusive thrombosis is a quantitative measure of in vivo vascular damage.

## Introduction

Thrombotic cardiovascular diseases are the most common cause of death and disability in the developed world [1–4]. The vessel wall is a complex system that includes multiple cell types and is influenced by multiple extrinsic factors including shear stress, circulating blood cells, hormones and cytokines, as well as the antioxidant status of the vessel wall [5,6]. Therefore, in vitro experiments cannot replicate this complex environment, so in vivo studies of animal models are critical to allow better understanding of mechanisms involved in thrombotic disorders [7–9]. Such studies mimic human pathology and may provide important mechanistic information related to discovery of new pathways and drugs, as well as important details characterizing drug effects.

Ferric chloride (FeCl_3_) induced vascular injury is a widely used model of thrombosis [10,11]. This agent, applied to the outer aspect of arteries induces oxidative damage to vascular cells [12,13], with loss of endothelial cell protection from circulating platelets and components of the coagulation cascade. This model is simple and sensitive to both anticoagulant and anti-platelets drugs, and has been performed on carotid and femoral arteries, jugular veins, and mesenteric and cremasteric arterioles and venules in mice, rats, guinea pigs and rabbits [12–19]. In this model FeCl_3_ is applied to the topical surface of an intact vessel, triggering vascular wall injury and denudation of the endothelium via a mechanism involving the generation of reactive oxygen species [4,14]. A recent study suggested that FeCl_3_-induced vascular injury was erythrocyte-dependent, requiring hemolysis and hemoglobin oxidation for endothelial denudation [20,21]. One measurable parameter in this model is the elapsed time from injury to complete vessel occlusion, measured as blood flow cessation by Doppler flowmeter or under direct observation with intravital microscopy [13–15]. A range of times between 5 and 30 min has been reported in different studies in C57Bl6 mice [12–15,22], suggesting that FeCl_3_ concentrations, types of anesthesia, surgical techniques, mouse age and genomic background, method of measuring blood flow, and other environmental variables have significant effects in this model. This wide variability may be acceptable for a single study, but it makes it very difficult to compare studies from different groups and may make detection of subtle differences difficult. In our recent serial studies of the role of the type 2 scavenger receptor CD36 in thrombosis associated with diet-induced atherosclerosis we have refined the traditional FeCl_3_-induced carotid artery model making the data highly reproducible with lower variation [12–15,22,23]. This paper will describe and share our skills and report different FeCl_3_ concentration or different treating time induced different redox injury on thrombosis.

## Materials and methods

All procedures and manipulations of animals have been approved by Institutional animal care and use committees (IACUC) of The Cleveland Clinic in accordance with the United States Public Health Service *Policy on the Humane Care and Use of Animals*, and the NIH *Guide for the Care and Use of Laboratory Animals*.

### Equipment and reagents

Tools for mouse surgery are available from Fine Science Tools (FST). Minimum necessary tools include: dissection scissor (14502-14), forceps (11018-12) for cutting skin, scissor (14519-14) and forceps (11050-10) for dissecting soft tissue, hemostatic forceps (13021-12), fine needle holders (12061-10), fine tip forceps [Dumont #5 (11 cm) 11254-20 and Dumont #5XL (15 cm) 11253-10], 3/8 curve needle, 4-0 silk suture, 6-0 silk suture, and a hook (10062-12). A black plastic coffee stir tube, Whatman #1 filter paper cut into 1×2 mm pieces, 15-cm tissue culture lids, surgical tape, a dissecting microscope (or loupes with at least 4X magnifications), heating pad with rectal temperature monitor probe, and hair cutter were also used. For real time observation of thrombosis we used a Leica DMLFS fluorescent microscope with an attached Gibraltar Platform (EXFO, Quebec, Canada) and water immersion objectives at 10x, 20x and 60x magnifications. Video imaging was performed using a QImaging Retigo Exi 12-bit mono digital camera (Surrey, Canada) and Streampix version 3.17.2 software (Norpix, Montreal, Canada). Ketamine (Ketaset 100 mg/ml, Fort Dodge Animal Health, IA, USA) and xylazine (100 mg/ml, Hospira, IL, USA) were used for anesthesia and rhodamine 6G (Sigma 252433-1G, 0.5 mg/ml in saline, 0.2 μm filtered) was injected intravenously used to label blood components. A stock solution of 30% FeCl_3_ (Anhydrous, Sigma 12321) in water was prepared and diluted prior to use at final concentrations of 2.5%, 5%, 7.5%, 10% and 12.5%.

### Arterial injury protocol

**Fig. 1:**
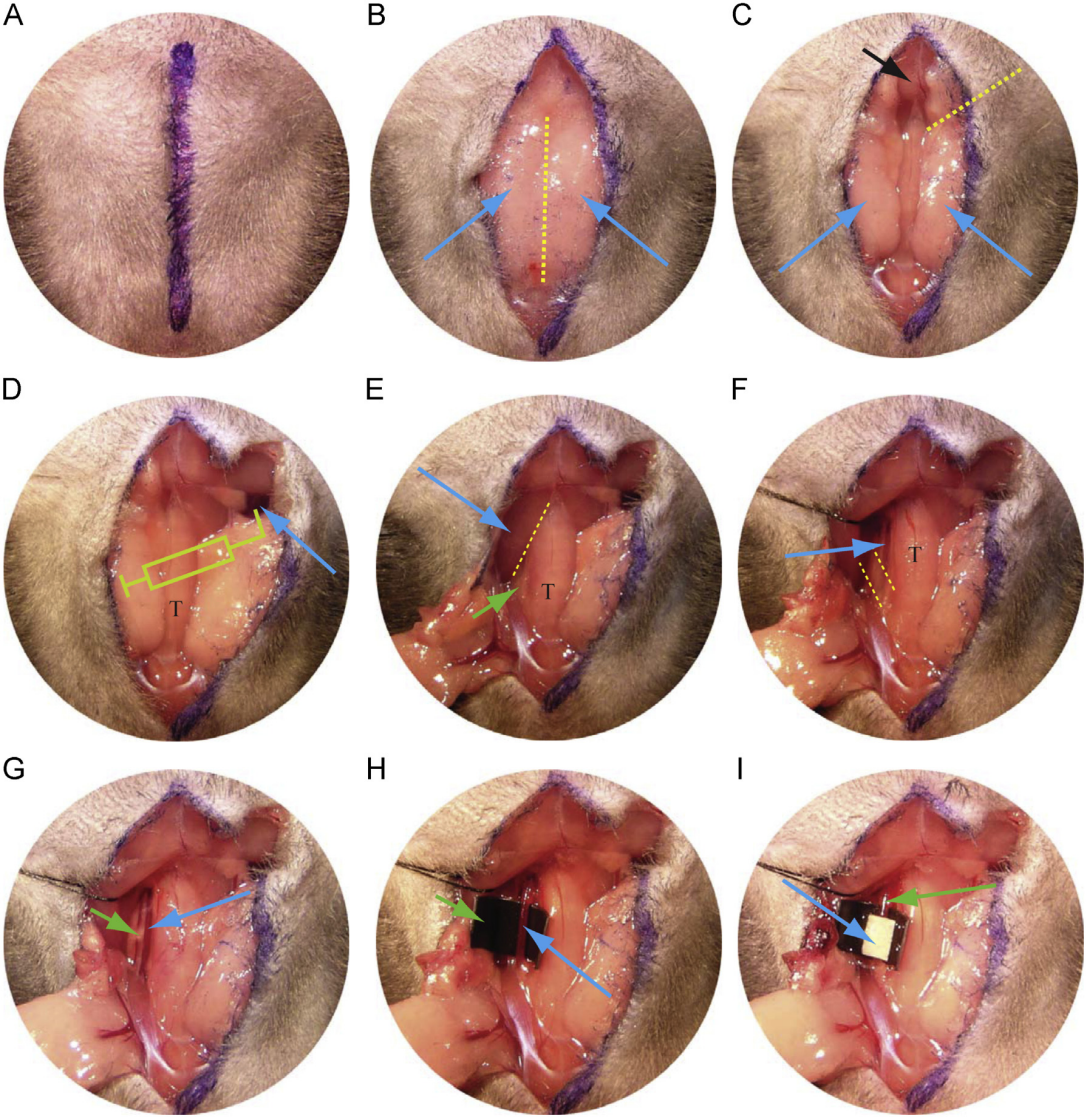
Procedures to expose the jugular vein, injection of rhodamine 6G flurescence dye into the circulation system and exposure of left carotid artery. T indicates trachea. See text for details.

1. Eight to twelve weeks old C57Bl6 mice are anesthetized with ketamine (100 mg/kg)/xylazine (10 mg/kg) via intraperitoneal injection. This anesthetic method facilitates manipulation of the animal during surgery and during transfer from surgical bench to the microscope stage.
2. Fur on the neck and upper chest is removed with a small animal electric clipper. Dipilatory cream is not used.
3. Mice are secured in the supine position on the lid of 15 cm tissue culture plate using tape (about 10 cm) to secure hind limbs and lower body, and two pieces of tape (2 cm×0.5 cm) to secure the forelegs. A 4**-**0 suture is wrapped around the incisors to keep the neck straight.
4. The mouse is placed with head towards the operator under a surgical light source. A midline incision is made through the skin from sternum to chin as shown in the line drawn in Fig. 1A.
5. The structures visualized first under the skin are the submaxillary glands (Fig. 1B and D, blue arrows) which are linked by a thin fascia (dotted line). This fascia can be dully dissected with a hemostatic forceps exposing the manubrium (black arrow in Fig. 1C) and the trachea (T in Fig. 1D).
6. The soft tissue seen to the right of the black arrow in Fig. 1C and the skin are held together with a forceps and a dissection forceps is then inserted under the fascia toward the 2 o'clock position (yellow dotted line in Fig. 1C) which is the position of the clavicle. The fascia is then freed from the jugular vein by opening the forceps.
7. The fascia, soft tissue and skin along the yellow dotted line in Fig. 1C are cut to expose the right jugular vein (Fig. 1D, arrow).
8. 100 μl of rhodamine 6G solution (or other drugs or fluorescent free radical indicators) is injected into the right jugular vein to label platelets using a 1 cc syringe with a fresh 30G needle. It is important to flush the needle with the solution to make sure that no air bubbles remain. To facilitate injection the needle can be bent 2–3 mm from tip to a 90° angle, which will make the tip parallel to the long axis of jugular vein (Fig. 1D). To stabilize the syringe and keep the needle in position the syringe is held in one hand and the needle with a forceps in the other during the injection. After injection, the site is held with the forceps, the vessel is clamped with a dissection forceps, and the vein is ligated with a 6-0 suture. It is important to perform the rhodamine injection before exposing the left carotid artery (CA) to prevent adventitial CA staining by the fluorescent dye if leakage or bleeding occurred during or after injection.
9. The soft tissue and fascia around the left submaxillary gland is then dully dissected and pulled toward the 7 o'clock position (Fig. 1E) to expose the left sternocleidomastoid muscle (blue arrow).
10. The fascia (Fig. 1E, dotted line) between the left sternocleidomastoid muscle and the omohyoid muscle or the sternohyoid muscle (Fig. 1E, green arrow) located to the left of the trachea is then dully dissected with a hemostatic forceps.
11. A needle with 6-0 silk suture (about 15 cm long) is passed under the sternocleidomastoid muscle at the level indicated by blue arrow in Fig. 1E and pulled laterally toward the 10 o'clock position to expose the common CA along or under the sternohyoid muscle and/or omohyoid muscle. This procedure must be performed with care to avoid injuring the left jugular vein, which is located outside of the sternocleidomastoid muscle.
12. The CA is seen after pulling away the sternocleidomastoid muscle (Fig. 1F arrow), which is accompanied by the vagus nerve, the white structure seen in Fig. 1G (green arrow). The very thin omohyoid muscle seen between the yellow dotted lines in Fig. 1F is removed if it covers the CA.
13. The hemostatic forceps are used to dissect the soft tissue around the CA without touching the CA, and then fine tip forceps are used to pick up the lateral fascia around the CA while avoiding the vagus nerve and CA. Another fine tip forcep is then used to punch a hole between the CA and vagus nerve. A hook is passed through the hole and the CA gently lifted so that a fine tip forcep can be placed under the CA. The hook and forceps are then in opposite directions along the CA to strip adventitial soft tissue.
14. To block background fluorescence, a black plastic coffee stirrer is cut into a 3 mm piece, pressed and cut longitudinally to yield two pieces of “U” shape plastic. One piece is inserted with forceps under the CA while gently lifting the CA with the hook (Fig. 1H).
15. To avoid drying of CA, 2–3 drops of saline are applied to the surgical field and the mouse and plate lid are transferred together to the microscope stage and the 10x water lens is placed in the appropriate position.
16. Since mechanical trauma can directly injure the endothelium and lead to thrombus formation [24] it is necessary to observe and record vessel images to confirm that no surgical injury happens to the vessel prior to the FeCl_3_ injury. It is also necessary to confirm that there is no remaining tissue around the CA, which may form a barrier and attenuate the effect of FeCl_3_-induced injury.
17. A paper towel is folded to make a thin and fine tip and used to carefully blot the saline around the CA (avoid touching CA) as well as in other places in the surgical field. This is very important to avoid dilution of the used FeCl_3_ solution.
18. Using a fine tip forceps to pick up a piece of filter paper (1×2 mm) saturated with FeCl_3_ solution and place directly on the CA for timed points. Then the filter paper is removed from the CA and rinsed with saline. To aid visualization of the blood stream the filter paper is placed toward the distal end of the exposed CA (Fig. 1I) making sure to leave a short segment of the proximal site (green arrow in Fig. 1I) for observing late phase blood flow.

### Observation of thrombi formation and the image record

Thrombus formation is identified by accumulation of the fluorescent platelets, which is observed in real-time recording video images or under microscope. Typically, we record video for 10 s every minute for the first 10 min and then 10 s every other minute. Make sure to note the video frame number at each time point for later analysis. After injury the vessel is scanned from distal to proximal sites of the exposed segments to provide a complete view of the initial injury, and then focus on the interest area for further imaging. When the thrombus occupies the entire segment it becomes difficult to identify blood flow in the thrombi. To solve this issue the imaging is moved to the proximal un-injured site (Fig. 1I, green arrow). In this site, blood flow should be still clearly identified. The end points of the experiment are: when blood flow has ceased for >30 s, or if occlusion is not seen after 30 min, the time is recorded as 30 min for statistical comparisons.

## Statistical analysis

One-way ANOVA (Bonferroni/Dunn) is used to determine the differences among groups. Unpaired *t* test is used to determine differences between groups. Data are represented as mean±SEM, and *P*<0.05 is considered significant.

## Results and discussion

Animal models of thrombosis not only provide valuable information about the mechanisms of thrombosis, but also are a valuable tool for pre-clinical evaluation of compounds that may be used for treatment and prevention of the atherothrombotic diseases [10,16,21,25]. Although the FeCl_3_ induced vessel injury model is a well-accepted tool for studying thrombosis, the mechanism of FeCl_3_ induced vascular lesion and the determinants initiating thrombus formation remains incompletely understood. One established concept is that FeCl_3_ mediated oxidation induces endothelial cell denudation and subsequent exposure of the subendothelial matrix to the blood stream, thus activating the coagulating system [4,21].

We made several refinements to the FeCl_3_ model to enhance its utility and reproducibility. These include: (1) exposing the CA sufficiently to provide enough length (∼5 mm) to allow application of the filter paper while leaving space to clearly observe blood flow by intravital microscopy; (2) stripping the adventitial soft tissue cleanly around the CA to allow the filter paper to contact the vessel wall directly and produce an even injury; (3) underlying the CA with a “U” shaped piece of black plastic to separate it from surrounding tissue, prevent FeCl_3_ diffusion, and block background fluorescence. This strategy is very important when using this model to examine vessel wall reactive oxygen species formation by injection of free radical indicator into the blood system [26]; (4) directly injecting a fluorescence dye into the jugular vein to label platelets in vivo. This does not affect platelet count or function, and obviates the need to sacrifice additional animals as platelet donors.

### Dose dependence of FeCl_3_-induced vessel occlusion time

**Fig. 2:**
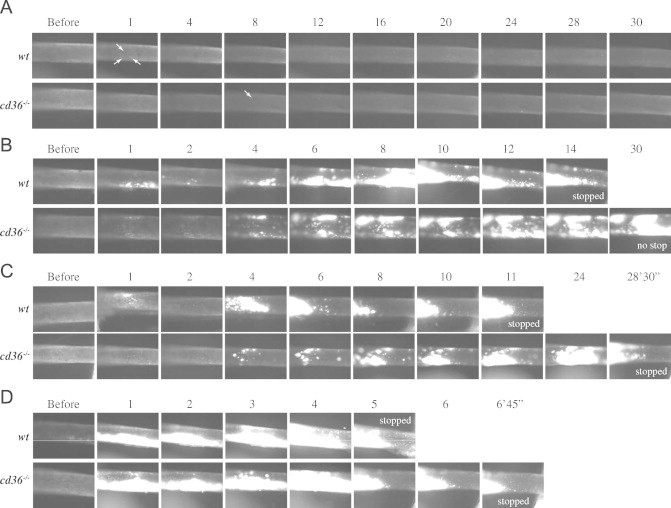
Effect of different concentration of FeCl_3_ on thrombosis. Representative video images of thrombi formation in carotid artery (CA) treated with different concentration of FeCl_3_, 2.5% (A), 5% (B), 7.5% (C), and 10% (D) are shown. Blood flow was from right to left. “Before” indicates images taken immediately prior to FeCl_3_ treatment. Numbers indicate time in minutes after removing the filter paper. Arrows in A indicate the tiny thrombi which were first observed.

We previously have reported that deletion of *cd36* prolonged vessel occlusion times [13]. We now used *wt* and *cd36* null mice, which are unable to bind and respond to oxidized lipoprotein particles and apoptotic cell particles, as tools to compare different concentration of FeCl_3_ on thrombosis. No thrombi were observed in the absence of FeCl_3_ application, showing that the surgical procedures did not induce vascular injury (Fig. 2). Treatment of CA with 2.5% FeCl_3_ for 1 min immediately induced formation of scattered tiny thrombi in *wt* mice (Fig. 2A, column 1 min, arrows). With time, the thrombi became larger, but at 30 min those were not large enough to impact blood flow. There were no thrombi formed in 2 of the 3 *cd36* null mice and only 4 tiny thrombi were observed (starting at 8 min) in the third mouse.

Treatment with 5% FeCl_3_ for 1 min immediately induced thrombi formation in *wt* and *cd36* null mice, and the border of the injured area was easy to identify (not shown). The thrombi formed initially were smaller in *cd36* null mice than in *wt* mice (Fig. 2B). These initial thrombi were washed away by the blood stream, so fewer thrombi were seen at 2 min. Thrombi started to enlarge 3–4 min after removing the filter paper and these later forming thrombi were stable and usually did not wash away. The thrombi in *wt* mice formed faster and were larger than those in *cd36* null mice; cessation of blood flow was seen in 3 of the 5 *wt* mice with an average vessel occlusion time of 23 min while none of the *cd36* null mice showed flow cessation within the 30 min of observation.

**Fig. 3:**
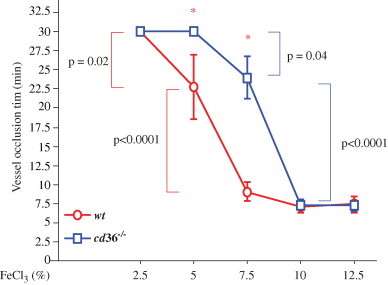
Vessel occlusion times after different concentrations of FeCl_3_ treatment. Mice were treated as in Fig. 2 and occlusion time assessed by intravital microscopy. *p<0.01 *wt* vs. *cd36* null at the same concentration of FeCl_3_. One-way ANOVA Bonferroni/Dunn was used to determine the differences among groups. Data are represented as mean±SEM. *N*=3 for 2.5% FeCl_3_ treated *wt* and *cd36* null mice; *n*=4 for 5% FeCl_3_ treated *cd36* null mice; and *n*=5 in all other conditions.

Treatment of the vessels with 7.5% FeCl_3_ induced more rapid increase in the size of thrombi in *wt* mice, but had no obvious difference in *cd36* null mice (Fig. 2C). As reported previously [27], this concentration of FeCl_3_ induced vessel occlusion in both types of mice; however, the vessel occlusion time was significantly elongated in *cd36* null mice. When the FeCl_3_ concentration was 10% or greater the vessel wall oxidation injury was presumably maximal (Fig. 2D) and no differences in occlusion time were seen between the two types of mice. Fig. 3 shows vessel occlusion time plotted as a function of FeCl_3_ and demonstrates a shift to the right of the response curve in *cd36* null mice compared to *wt*, suggesting a pro-thrombotic effect of CD36. These data are also consistent with our recent data that *cd36* deletion induced an antioxidant effect on the vessel wall [14].

When thrombi become larger and cover the entire area of injury, it becomes difficult to observe flow (e.g. Fig. 2B, 8 min time point in *wt*, and online video). This may be the reason why some researchers think that this method is less sensitive than measurement of blood flow by transonic flowmeter. By surgically exposing the CA over a sufficient length and placing the filter paper close to the distal site (Fig. 1I) we were able to observe and record the normal area proximal to the site of injury as shown in Fig. 1I (arrow) and Fig. 2C (e.g. 6–11 min in 7.5% FeCl_3_ treated *wt* mouse), and blood flow can be identified clearly. When blood flow ceases, numerous larger cells (leukocytes) start to adhere to the vessel wall at the proximal site of the thrombi; therefore, large cell accumulation can be used as a secondary marker for oxidatve vascular damage. Flow usually stopped within 2 min of the appearance of large cells (see online video for more information). In our studies we recorded the time when blood flow completely stopped. This may differ from some studies using a transonic flow probe where background noise makes it difficult to determine the flow precisely, especially when it falls to less than 10% of baseline [28].

Supplementary material related to this article can be found online at doi:10.1016/j.redox.2012.11.001.

The following is the Supplementary material related to this article Video 1

Video 1 Online video: Representative video of thrombi formation after 7.5% FeCl_3_ treatment for 1 min is shown. Blood flow was from right (heart) to left (head).

### The duration of FeCl_3_ treatment correlates with time to vessel occlusion

**Fig. 4:**
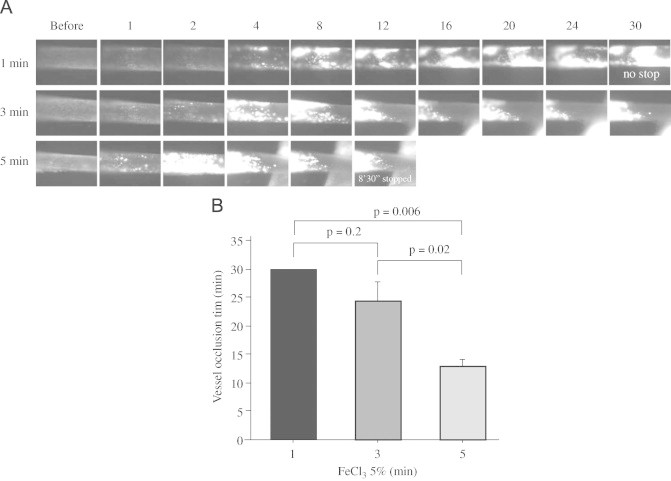
Duration of FeCl_3_ treatment on thrombosis. (A) Representative video images of thrombi in CA exposed to 5% FeCl_3_ for 1, 3 or 5 min. “Before” indicates before FeCl_3_ treatment. Numbers indicate time in minutes after removing the filter paper. (B) Statistical analysis of vessel occlusion time. *N*=3 for 1 min and *n*=5 for 3 and 5 min treatments. One-way ANOVA (Bonferroni/Dunn) was used to determine the differences among groups. Data are represented as mean±SEM.

Published studies have used a range of exposure times of FeCl_3_ treatment, making comparisons among studies difficult. To determine the effect of varying exposure times in this model system we treated *cd36* null mice with 5% FeCl_3_ for 1, 3 and 5 min. As shown in Fig. 4, increasing the duration of FeCl_3_ exposure dramatically increased the rate of thrombus enlargement and shortened the time to blood flow cessation. We previously showed that *cd36* deletion protects VSMC from oxidant stress and reduces the amount of microparticles in the circulation after FeCl_3_ injury. We also showed that microparticles contribute to thrombosis by interacting with platelet CD36 [13]. Others have shown that FeCl_3_-injury induces release of endothelial cell-derived FeCl_3_-filled “buds” that are rich in tissue factor and may contribute to thrombosis [21], while VSMC-derived tissue factor can contribute to macrovascular thrombosis [29]. Our data suggest that the duration of FeCl_3_ exposure is an important component of the injury response in mouse models, perhaps by influencing the level of endothelial and VSMC injury and microparticles release after exposing the vessel to oxidative stress.

In summary, we have refined and standardized components of the FeCl_3_-indcued vascular injury model to produce a highly reproducible injury response where we can quantitate blood flow cessation accurately by intravital microscopy. This allowed us to generate reproducible results with low variance [4,12–15,22,30] to demonstrate FeCl_3_ dose and exposure duration, and hence oxidative insult, influences thrombosis in vivo.

## Contribution of authors

Dr. Li performed the experiments, data analysis and wrote the paper. Dr. McIntyre and Dr. Silverstein contributed to read and write the paper critically.

## Conflict of interest statement

The authors have no conflicts of interest related to this study.
